# In Vitro Stimulation of Multidrug Resistance-Associated Protein 2 Function Is Not Reproduced In Vivo in Rats

**DOI:** 10.3390/pharmaceutics10030125

**Published:** 2018-08-08

**Authors:** Ravindranath Reddy Gilibili, Vishwanath Kurawattimath, Bokka Venkata Murali, Yurong Lai, T. Thanga Mariappan, Hong Shen, Sagnik Chatterjee

**Affiliations:** 1 Pharmaceutical Candidate Optimization, Biocon Bristol-Myers Squibb R&D Center (BBRC), Syngene International Ltd., Bangalore 560100, India; ravindra.reddy@syngeneintl.com (R.R.G.); Vishwanath.Kurawattimath@syngeneintl.com (V.K.); Venkata.murali@syngeneintl.com (B.V.M.); Thanga.Mariappan@syngeneintl.com (T.T.M.); 2 Department of drug metabolism, Gilead Sciences Inc., Foster City, CA 94404, USA; Yurong.Lai@gilead.com; 3 Pharmaceutical Candidate Optimization, Bristol-Myers Squibb Company, 3551 Lawrenceville Road, Princeton, NJ 08540, USA; hong.shen1@bms.com

**Keywords:** ABCC2, MRP2, in vitro stimulation, drug transporter, coproporphyrin

## Abstract

Previously we reported that coproporphyrin-I (CP-I) is an optimal probe substrate for multidrug resistance-associated protein 2 (MRP2), and stimulation of MRP2-mediated transport is probe substrate-dependent. In the present investigation, we assessed if the in vitro stimulation is physiologically relevant. Similar to human MRP2 transport, CP-I was transported by rat Mrp2 in a typical Michaelis-Menten kinetics with apparent K_m_ and V_max_ values of 15 ± 6 µM and 161 ± 20 pmol/min/mg protein, respectively. In vivo Mrp2 functions were monitored by biliary and renal secretion of CP-I and its isomer CP-III, in bile-duct cannulated rats before and after treatment with mitoxantrone, progesterone, and verapamil. These compounds stimulated Mrp2-mediated CP-I transport in vitro. No significant increase in biliary or renal clearances, as well as in the cumulative amount of CP-I or CP-III eliminated in bile, were detected following treatment with the in vitro stimulators, indicating an in vitro to in vivo disconnect. In presence of 10 µM bilirubin, the in vitro stimulation was suppressed. We concluded that the in vitro stimulation of CP-I transport mediated by Mrp2 is not translatable in vivo, and proposed that the presence of endogenous compounds such as bilirubin in the liver may contribute to the in vitro to in vivo disconnect.

## 1. Introduction

It is becoming increasingly evident that multidrug resistance-associated protein 2 (MRP2/Mrp2, denoted by gene ABCC2/Abcc2) plays an important role in disposition and elimination to regulate pharmacokinetics and pharmacodynamic of xenobiotics [1,2]. MRP2 is located in the canalicular membrane of hepatocytes but also in the apical (luminal) membrane of enterocytes and renal proximal tubule cells [3]. In hepatocytes, MRP2 facilitates the efflux of glucuronide and glutathione conjugates of endobiotics, such as bilirubin conjugates and dianionic bile acids [4], as well as few anionic xenobiotics, such as methotrexate [5]. The inhibition of MRP2 activity is therefore important for drug hepatic disposition and elimination, and can cause potential drug-drug interactions (DDI) and hepatotoxicity [6]. For example, a two-fold increase in methotrexate plasma levels due to MRP2 polymorphism has been reported [7]. However, changes in MRP2 activity in vivo is usually associated with profound changes in liver exposure, and minor/no effect on plasma exposure, and therefore it usually remains undetected [8]. Recently, it was shown that atorvastatin liver concentration increases 1.64-fold, in the presence of metformin, an Mrp2 inhibitor, without affecting the plasma exposure in Sprague Dawley (SD) rats [9].

Interactions with MRP2 are commonly characterized in vitro, in an inverted membrane vesicle expressing MRP2/Mrp2 in the presence of ATP. Decrease or increase in ATP-dependent transport of probe substrates in the presence of an interacting compound suggest either inhibition or stimulation. To investigate if a compound is an inhibitor of MRP2/Mrp2, estradiol 17-β-glucuronide (E17βG) is widely used as a probe substrate [1]. However, many compounds such as indomethacin, diclofenac, and benzbromarone are reported to “modulate” the transport of E17βG [10,11,12,13]. Modulation refers to stimulation at lower concentrations of the compound, followed by inhibition at higher concentration, providing a “bell-shaped” curve in a percent (%) transport against interacting compound concentration plots. The compounds that impart similar profile to the transport of probe substrates are henceforth termed as modulators or stimulators interchangeably in this article. The presence of a modulation site, in addition to active transport site in the MRP2 protein, is proposed to explain the modulation and “bell-shaped” curve [10,14,15]. There are two manuscripts that explored the in vivo relevance of the in vitro stimulation of Mrp2, in rats. Heredi-Szabo et al. used indomethacin as a stimulator and reported that indomethacin modestly decreases the half-life (40%) and increases E17βG biliary clearance [16]. In addition, Ito et al. used benzyl penicillin as a stimulator and found that biliary clearance of glutathione (GSH) and bile flow were transiently enhanced (about four-fold for GSH and two-fold for bile flow) [17]. However, in vivo relevance of the substrate-dependence of in vitro MRP2 stimulation remains unknown. In addition, there are no reports of any clinical drug-drug interaction or toxicity that has been attributed to this stimulation. This raises questions on the in vivo relevance of in vitro stimulation of MRP2-mediated probe substrate transport.

Recently, we demonstrated that coproporphyrin-I (CP-I) is an optimal probe substrate for MRP2 [10]. CP-I is a byproduct of heme biosynthesis and its physiological role is not yet clear. The uptake of CP-I in MRP2-overexpressing membrane vesicles follows a typical Michaelis-Menten equation with a low K_m_ value of 7.7 µM. When CP-I is used as a MRP2 probe for in vitro characterization of MRP2 inhibition, a lower percentage of compounds were found to stimulate MRP2 mediated transport, compared to E17βG [10]. In our study using 97 compounds, we found 51 (53%) to stimulate MRP2-mediated E17βG transport, while only 8 out of 47 (17%) compounds stimulated the transport of CP-I, while 30 compounds were found to inhibit CP-I transport [10]. The results suggest that the stimulation appeared to be a probe substrate-dependent phenomenon, rather than an intrinsic property of the transporter. Furthermore, the stimulators shared the basic backbone structures of physiological steroids, which raises a question of potential in vivo physiological relevance of in vitro stimulation of MRP2-mediated CP-I transport [10].

The aim of the current study was to investigate whether the compounds that stimulated Mrp2-mediated CP-I transport in vitro, also stimulate Mrp2-mediated CP-I transport in vivo. Mrp2-mediated in vivo transport is appreciated by measuring biliary clearance of CP-I and its isomer CP-III in rats, in the presence of selected stimulators. The stimulation of CP-I transport in rat Mrp2 transfected membrane vesicles was confirmed and a mechanistic understanding for the perceived in vitro to in vivo disconnect put forward with potential in vitro evidence.

## 2. Materials and Methods

### 2.1. Materials/Chemicals

Mitoxantrone hydrochloride, coproporphyrin-I dihydrochloride (CP-I), progesterone, verapamil, ritonavir and testosterone were procured from Sigma-Aldrich (Sigma-Aldrich Chemie GmbH, Munich, Germany). d^4^-CP-I (15N^4^ deuterated) sodium bisulfate salt procured from Toronto Research Chemicals (Toronto, ON, Canada). MultiScreen Solvinert filter plates (0.45 μm, low binding hydrophilic polytetrafluoroethylene) were purchased from Millipore (Tullagreen, Ireland). High-performance liquid chromatography–grade methanol was purchased from Merck (Mumbai, India). Formic acid was purchased from Fluka (Fluka Chemie, GmbH, Munich, Germany) and Milli-Q water from Milli-Q system (Millipore SAS, Molsheim, France). Polyethylene-10 and -50 tubing was purchased from Smiths Medical (ASD incorporation, Dublin, OH, USA), and 22-gauge needles and syringes were purchased from Becton Dickinson India Pvt Ltd. (Bangalore, India). Rat Mrp2-expressing inside-out membrane vesicles (protein concentration 4 mg/mL) derived from Sf9 insect cells were in-house prepared. Reaction incubation plates (96-well, ultra-low attachment, polystyrene, flat bottom, clear), Filter plate, 96 well (0.25 mm Glass Fiber/1.2 µm PES) and assay plates (96 well, black, flat bottom, polystyrene) for fluorescence measurement purchased from Corning^®^ Costar^®^ (Kennebunk, ME, USA). Krebs-Henseleit buffer purchased from Bioreclamation IVT (Bangalore, India). Stock solutions were prepared in dimethyl sulphoxide and stored at −70 °C.

### 2.2. Animals

Male Sprague Dawley rats weighing 300 to 350 g (10–12 weeks of age) were obtained from Vivo Bio Tech Ltd., Hyderabad (India). All animal experiments were conducted in the animal research facility of Syngene International Ltd., Bangalore, India, after obtaining approval of the Institutional Animal Ethics Committee (Approval details: Title: Investigation of Metabolism and Elimination pathways of New Chemical Entities (NCEs) in rats; SYNGENE/IAEC/858/07-2017; Principal Investigator/Research Scholar: Mr. Vishwanath K M). The committee was registered for the Purpose of Control and Supervision on Experiments on Animals and accredited by Association for Assessment and Accreditation of Laboratory Animal Care International. The animals were fed a standard laboratory rodent diet (Tetragon Chemie Pvt. Ltd., Bangalore, India) and housed at room temperature (22 ± 3 °C) and relative humidity of 50 ± 20% on a 12-h light and dark cycle. Water was provided ad libitum throughout the study. An intravenous solution formulation of mitoxantrone (6 and 15 mg/mL) and verapamil (10 mg/mL) were formulated as a solution by using 100% saline. Progesterone (1 mg/mL) was formulated using a solution of dimethylacetamide: Solutol: PEG400: water (5:5:75:15% *v*/*v*).

### 2.3. Vesicular Transport Assay

The vesicular transport assay in membrane vesicles overexpressing rat Mrp2 was conducted using the methods reported previously [10]. Briefly, rat Mrp2 membrane vesicles were diluted to an appropriate concentration in buffer-A containing 50 mM MOPS-Tris (pH 7.0), 70 mM KCl, 7.5 mM MgCl_2_. Membrane vesicles (20 µL, 50 µg protein) were co-incubated with 0.5 µL of test substrates (CP-I) at 37 °C for 3 min. Then, the reaction was initiated by adding pre-warmed buffer-A (29.5 µL) premixed with 4 mM MgATP or 4 mM MgAMP and 2 mM glutathione. Following incubation for designated times at 37 °C on a rotary shaker (Innova 40, New Brunswick Scientific Co., Inc., Enfield, CT, USA) at 100 rpm, the reaction was stopped by adding 150 µL of cold wash with buffer-B containing 40 mM MOPS-Tris (pH 7.0), 70 mM KCl. The reaction mixture was then transferred into a pre-wet, 96-well filter plate which was placed onto a filtration device (FiltrEX™ 96-Well Filter Plates, Corning Technologies India Pvt Ltd., Pune, India), and filtered rapidly via a connected vacuum pump (MultiScreen^®^HTS Vacuum, Manifold, MA, USA). All wells were washed 5 times, each time with 200 μL of ice-cold wash buffer to remove excess CP-I. After the final wash, the filter plate was dried at room temperature for 1 h. Membrane vesicles were digested using an extraction solvent (100 µL of 0.5% SDS dissolved in milliQ water, and the plate was kept on a microplate shaker (VWR, Radnor, PA, USA) for 15 min at 230 rpm. Then, the filter plate was centrifuged (Eppendorf, Hauppauge, NY, USA) for 2 min at 2000 rpm along with a receiver plate attached to collect the filtrate. The filtrate was further used to quantify the CP-I levels using a fluorimeter. Fluorescence measurements were conducted with a microplate reader (SpectraMax^®^ M2e, molecular device, San Jose, CA, USA), using 401 and 595 nm as excitation and emission wavelengths, respectively. ATP-dependent net transport was calculated by subtracting the AMP values from those of ATP values. All experiments related to CP-I were conducted in reduced light to minimize florescent bleaching.

Mitoxantrone, testosterone, progesterone, and verapamil were selected to assess their modulatory effects on rat Mrp2-mediated CP-I transport in membrane vesicles. The final assay concentration of CP-I was 5 µM. The concentrations tested for each modulator were 1, 10, 50, 100, 250, 500, and 1000 µM, except verapamil (25, 50, and 100 µM).

### 2.4. In Vivo Studies with Bile Duct Cannulated Rats

Bile duct cannulation on Sprague-Dawley (SD) rats was conducted [18] in a cross-over study design. The freely moving bile duct cannulated rats were kept in metabolic cages. Following 48 h acclimatization, rats were dosed with saline (dosed in all the groups as a control at 1 mL/kg dose volume) and bile, then plasma and urine were collected at specified time points. Ten minutes after the last bile, plasma, and urine sample collection, the same rats were dosed with mitoxantrone, verapamil, and progesterone via the jugular vein as an intravenous infusion at 1 mL/kg dose volume by using a single syringe model ‘11’ Pico plus pump. The doses used were 6 and 15 mg/kg for mitoxantrone, 10 mg/kg for verapamil, and 1 mg/kg for progesterone. The blood samples (200 µL) were collected at 0.17, 0.25, 0.5, 0.75, 1, 1.25, 1.5, 2, 2.5, and 3 h and bile samples were collected in pre-weighed amber color tubes over 15 min intervals up to 1.5 h and over 30 min intervals up to 3 h after dosing mitoxantrone. The blood samples (200 µL) were collected at 0.17, 0.25, 0.5, 0.75, 1, 1.5, and 2 h and bile samples were collected in pre-weighed amber color tubes over 10 to 15 min intervals up to 1 h, and over 30 min intervals up to 2 h after dosing verapamil and progesterone. In addition, 0–3 h urine samples and terminal liver samples were collected from 3 rats using serial sampling. Blood was collected through the jugular vein in tubes containing 2% *w*/*v* potassium ethylenediaminetetraacetic acid solution, and bile was collected in pre-weighed amber tubes, rats were euthanized using carbon dioxide inhalation, liver samples were harvested and homogenized using 4 volumes of water. The bile, plasma, and liver homogenate samples were stored at −80 °C until further analysis.

### 2.5. Liver Free Fraction Determination for Mitoxantrone, Verapamil and Progesterone

The free fraction of the test compounds (Mitoxantrone, Verapamil, and Progesterone) in liver was determined using a rapid equilibrium dialysis (RED) device. Ritonavir spiked in human plasma at 2 µM concentration was used as a positive control for this purpose. The fu (free fraction) was calculated using the formula given by Riccardi et al. [19]. Briefly, blank liver homogenates were prepared at two different set of dilutions (50-times and 100-times) in 100 mM phosphate buffer using homogenizer (POLYTRON^®^, PT4000, Kinematica AG, Luzern, Switzerland). The test compounds were then individually spiked into these liver homogenates to achieve a target concentration of 10 µM. RED device (Thermo Fisher Scientific, Hudson, NH, USA) was set up as per the manufacturer’s instructions (Instructions, RED Device inserts, 89809, Thermo Scientific, Marietta, OH, USA). An aliquot of 200 µL spiked samples were added into the donor side in triplicate, and 350 µL of 100 mM potassium phosphate buffer into the respective receiver side and allowed to equilibrate for 6 h in a HEPA class 100, CO_2_ incubator (Steri-cycle, Model 370, Thermo Scientific, Marietta, OH, USA) maintained at 37 °C with 5% CO_2_. At the end of the incubation, 25 µL incubate from the donor side was aspirated and mixed with 25 µL of 100 mM phosphate buffer; similarly, 25 µL from receiver side was mixed with 25 µL of respective control liver homogenates in a collection plate. Then 150 µL of acetonitrile containing internal standard (Propranolol at 150 nM) was added to the plate and mixed with a multi-channel pipette (Thermo Scientific, FINNPIPETTE F, MH-14040, and Finland). The contents were then transferred to Solvinert filter plate (Merck Millipore Ltd., Tullagreen, Ireland) fitted with 1 mL capacity 96-well collection plate (Waters Corporation, Milford, MA, USA). The plate was centrifuged at 3220× *g* for 5 min and the filtrate was injected on LC-MS/MS. Analyte concentrations in study samples were then obtained from the calibration standards prepared similarly, and by plotting the analyte to internal standard peak area ratios against respective analyte concentrations. Analyst version 1.6.2 was used for system control and data processing. The reported % fu was calculated as average of fu obtained from both 50-times and 100-times dilutions.

### 2.6. LC-MS/MS Method

LC-MS/MS method was developed with adequate sensitivity for the quantification of CP-I, CP-III, mitoxantrone, verapamil, and progesterone using a UPLC system (Waters Corporation, Milford, MA, USA) coupled with a Triple Quad 5500 System (AB Sciex, Framingham, MA, USA), operated in positive electrospray ionization. A combined internal standard solution consisting of d^4^-CP-I (15N^4^ deuterated) sodium bisulfate salt and ritonavir was used during the analysis. d^4^-CP-I was used as internal standard in the analysis of CP-I and CP-III, whereas ritonavir was used as internal standard (IS) for the analysis of progesterone, mitoxantrone, and verapamil. The analytes and internal standards were separated on an Ace excel 2 C18-150 × 2.1 mm column (Advanced Chromatography Technologies Ltd., Aberdeen, Scotland) using a binary gradient elution with 10 mM ammonium formate in water with 0.1% formic acid (solvent-A); and acetonitrile with 0.1% formic acid (solvent-B). The gradient started with 40% solvent B and maintained for 0.5 min; in 2.5 min, solvent B was increased from 40 to 55% and then to 95% in 2.8 min and remained constant at 95% solvent B for 1.10 min. Then in 3.80 min solvent B was decreased from 95 to 40%, and it remained constant for 0.2 min, accounting to a total gradient time of 4 min. The flow rate was set at 0.5 mL/min. The mass spectrometric conditions adopted in the analysis were as follows: Capillary voltage, 5500 V; drying gas temperature, 450 °C; and nebulizer gas pressure, 50 psi (both nebulizer and drying gas were high-purity nitrogen). The MRMs monitored for the compounds were as follows: CP-I (655.5 → 596.4), CP-III (655.5 → 596.4), mitoxantrone (455.1 → 358.3), verapamil (455.2 → 165.1), progesterone (315.3 → 297.5), d4-CP-I (659.3 → 600.3), and ritonavir (721.2 → 296.2). Respective analyte concentrations in study samples were obtained from the calibration curves, plotted as analyte to internal standard peak area ratios against respective analyte concentrations. Analyst version 1.6.2 was used for system control and data processing.

A total of 15 calibration standards were prepared in 1% bovine serum albumin (BSA) by serial dilution. All the bile samples and urine samples were diluted in 1% BSA (~10 fold) prior to analysis. Then 100 µL of the sample was quenched with 800 µL of acidified acetonitrile (1% formic acid) containing IS. The samples were vortexed on a spinix mixer (The Scientific House, Chennai, India), and centrifuged in a refrigerated centrifuge (Minispin plus, Eppendorf AG-22331, Hamburg, Germany) at 14,000× *g*, 4 °C for 5 min. A supernatant of approximately 150 µL was transferred into a 96-well plate and subjected to LC-MS/MS analysis. 

### 2.7. Pharmacokinetic Analysis

CP-I and CP-III area under the plasma concentration-time profiles (AUC) were calculated by non-compartmental analysis and mixed log linear method using Kinetica software (Version 5.5.1; Thermo Electron Corporation, Waltham, MA, USA). Cumulative amounts of CP-I and CP-III were calculated by using Microsoft excel. The biliary (CLb) and renal (CLr) clearances of CP-I and CP-III were estimated by the following equations:

$${\mathrm{CL}}_{b}=\frac{X_{b}}{\mathrm{AUC}}\;$$

$${\mathrm{CL}}_{r}=\frac{X_{r}}{\mathrm{AUC}}\;$$

where, X_b_ and X_r_ are cumulative amounts of CP-I and CP-III excreted in bile and urine, respectively.

### 2.8. Statistical Analysis

Statistical analysis was performed using the GraphPad Prism program version 5.02 (GraphPad software, San Diego, CA, USA). Mean and standard deviations are calculated in Microsoft Excel 2013. Student t test was conducted to compare the cumulative amount of CP-I and CP-III obtained at each time point when administered saline and different modulators (mitoxantrone, verapamil, and progesterone); *p* < 0.05 was regarded as significant. No statistical test was conducted in in vitro experiments, as they represent triplicate data from only one experiment.

## 3. Results

### 3.1. Mrp2-Mediated CP-I Transport in Membrane Vesicles

**Figure 1:**
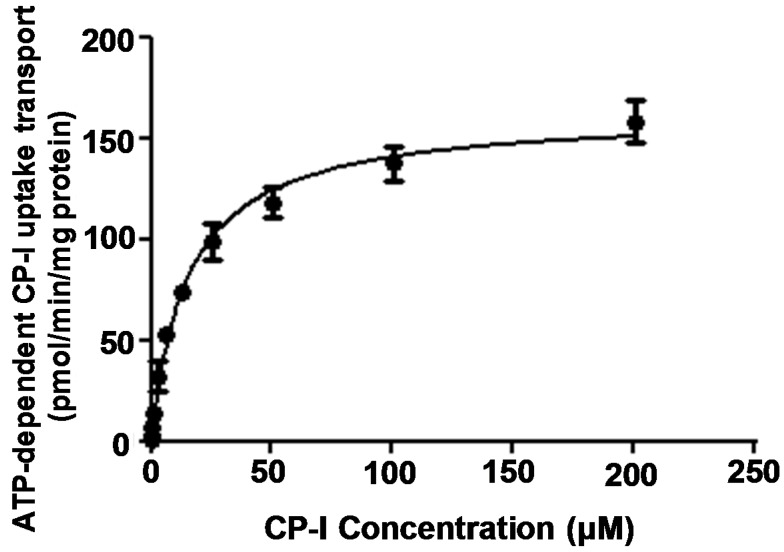
Concentration-dependent uptake transport of coproporphyrin-I (CP-I) in rat multidrug resistance-associated protein 2 (Mrp2) membrane vesicles. Rat Mrp2 membrane vesicles were incubated with CP-I at 37 °C for 15 min. ATP-dependent CP-I uptake is measured by subtracting the uptake in the presence of AMP from that of ATP. For more experimental details refer to Section 2.3. All data values are presented as mean and SD of a single experiment performed in triplicate wells.

In time linearity studies, Mrp2-mediated CP-I transport was linear with time up to 30 min, and a 15 min time point was used for all incubations unless specified. The transport kinetics of CP-I were then characterized by assessing concentration-dependent transport of CP-I in membrane vesicles overexpressing rat Mrp2 protein. ATP over AMP ratio of CP-I uptake is 23, with negligible uptake in the presence of AMP. As depicted in Figure 1, the concentration-dependent CP-I uptake appeared to follow a hyperbolic relationship. The kinetic curve could be adequately modelled by the standard Michaelis-Menten equation. The apparent K_m_ and V_max_ values (mean ± SD) for CP-I were 15 ± 6 µM and 161 ± 20 pmol/min/mg protein, respectively.

**Figure 2:**
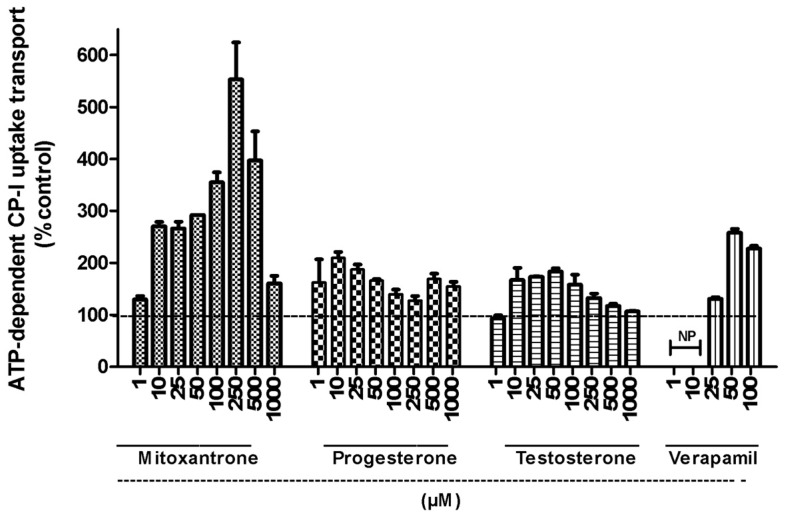
Effect of various modulators (mitoxantrone, progesterone, testosterone, and verapamil) on the uptake of CP-I in rat Mrp2 membrane vesicles. Rat Mrp2-mediated CP-I transport was measured in inside-out membrane vesicles. Mrp2 vesicle protein (50 µg/well) were preincubated with modulators at above reported concentrations at 37 °C and reaction was started by adding CP-I (5 µM) and incubated for 15 min. NP; not performed. For more on experimental details refer to Section 2.3. Dotted line represents basal level (100%) of CP-I uptake in control wells in the presence of ATP with no modulators added. All data values presented as mean and SD of single experiment performed in triplicate wells. In the inset, same data set is represented in curve to show the bell shape of the concentration-dependent stimulation and inhibition (also referred as modulation in the text).

**Figure 3:**
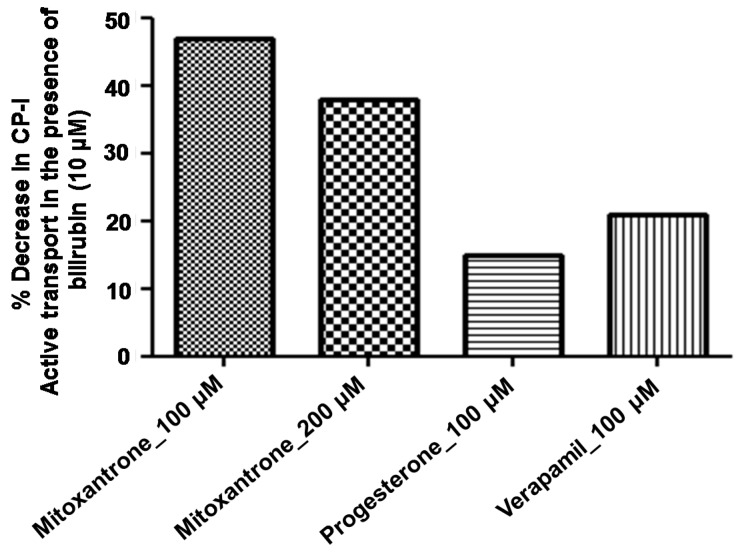
Effect of bilirubin on the transport of CP-I using rat Mrp2 vesicles in the presence and absence of modulators. Rat Mrp2 vesicles (50 µg/well) were preincubated with and without bilirubin (10 µM) and modulators for 3 min at 37 °C. % Net decrease in ATP-dependent CP-I uptake in presence of bilirubin was calculated by taking CP-I uptake values in presence of modulators as control. All data values presented as mean of single experiment performed in duplicate wells. The duplicate values are: 54%, and 40% (mitoxantrone 100 µM), 34% and 42% (mitoxantrone 200 µM), 17% and 12% (progesterone 100 µM), and 21, and 21% (verapamil 100 µM).

Figure 2 shows the concentration-dependent effect of various modulators (mitoxantrone, progesterone, testosterone, and verapamil) on the uptake of CP-I in rat Mrp2-overexpressing membrane vesicles. These modulators were selected based on the previous results found with human MRP2 vesicles [10]. As expected, all four modulators showed increased uptake of CP-I (stimulation) up to a specific concentrations and then showed inhibition of uptake at higher concentrations in rat Mrp2 vesicles, leading to a bell-shaped curve. This trend of stimulation and inhibition is common for all the four modulators, although at different concentration ranges, which agree with the results observed in human MRP2 vesicles (Gilibili et al., 2017). The interactions between CP-I uptake in rat Mrp2 vesicles and the above stimulators were further conducted in the presence of 10 µM bilirubin. As shown in Figure 3, the presence of bilirubin suppressed the stimulation of CP-I transport in rat Mrp2 vesicles by 200 µM mitoxantrone, 100 µM progesterone, and 100 µM verapamil. The suppression reached a maximum (about 50%) at 100 µM mitoxantrone.

### 3.2. Plasma Level and Biliary Secretion of Endogenous CP-I and CP-III in Rats

**Figure 4:**
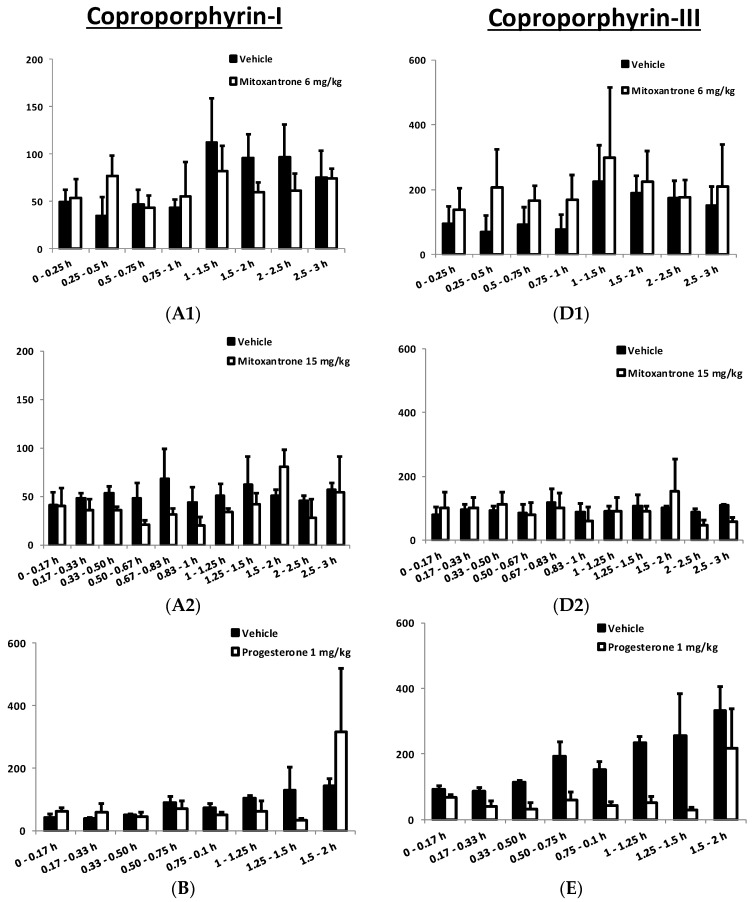

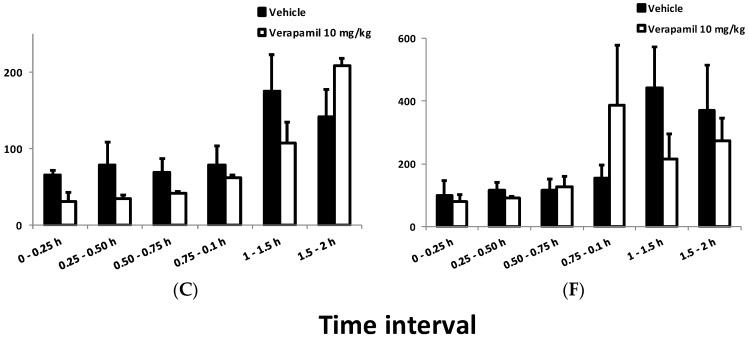
Amount of CP-I and CP-III excreted in bile at different time intervals in the presence (□) and absence (■) of modulators. Biliary excretion profile of endogenous CP-I and CP-III in male Sprague Dawley (SD) rats following intravenous administration of various modulators. All animals received saline (0.9% NaCl) first then modulator after collection of bile at designated time points. (**A1**,**D1**,**A2**,**D2**); represent biliary excretion profile of CP-I and CP-III in male SD rats treated with mitoxantrone at 6 and 15 mg/kg, respectively. (**B**,**E**) represents biliary excretion profile of CP-I and CP-III in male SD rats treated with progesterone at 1 mg/kg. (**C**,**F**) represents biliary excretion profile of CP-I and CP-III in male SD rats treated with verapamil at 10 mg/kg. All the data values presented in mean and SD of single experiment performed in three animals.

To understand whether the observed in vitro effect of modulators on transport of CP-I is translated in in vivo systems, studies were conducted using bile duct cannulated rats. In this model, the modulators were administered as an infusion through the jugular vein. Plasma and bile levels of CP-I and CP-III were measured at different time intervals during 2 h (for verapamil and progesterone) and 3 h (for mitoxantrone) pre- and post-dose treatment with modulators. The basal levels of CP-I and CP-III excreted in 2 h were found to be about 600 and 1200 pmoles in rat bile, respectively. The plasma AUCs (0–2 h) were found to be about 1.2 and 9.5 nM.h for CP-I and CP-III, respectively. Figure 4 shows the amount CP-I and -III excreted in bile, in the presence and absence of the modulators at different time durations. As can be observed from Figure 4, there was a significant increase in biliary excretion of CP-I and III between 0.25 and 0.5 h following dosing of mitoxantrone 6 mg/kg (Figure 4A1,D1). However, the same changes were not observed with 15 mg/kg mitoxantrone (Figure 4A2,D2). At the higher dose of mitoxantrone (15 mg/kg), the biliary secretion of CP-I was decreased from 0.33 to 1.25 h. In contrast, with administration of progesterone at 1 mg/kg, a significant decrease in biliary secretion of CP-III from 0.17 to 1.5 h was observed. A similar decrease of CP-I biliary clearance was also observed from 0 to 0.75 h, following 10 mg/kg verapamil treatment.

The biliary (Table 1) and renal clearances (Supplementary Table S2) over the 2 or 3 h time periods, with and without the modulators treatment, were calculated as described in materials and methods. The average biliary clearance of CP-I is four-fold higher than CP-III in the control experiments, while urinary clearance of CP-III is three-fold higher than CP-I. No significant increase in biliary or urinary clearance of CP-I or CP-III is observed at pre- and post-dosing with modulators (*p* > 0.05). Supplementary Table S1 lists the cumulative amount of CP-I and CP-III excreted in bile, in the 2 or 3 h time interval. The cumulative amount of CP-I excreted in bile and the plasma AUC of CP-I were found to be similar in the presence and absence of all the three modulators. A similar trend was also observed in CP-III levels.

The total and free liver concentrations of the stimulators followed by i.v infusion were determined. The liver concentrations of mitoxantrone (at 15 mg/kg), progesterone, and verapamil were 950, 197, and 1040 µM, respectively, resulting in free liver concentration (C_max_, unbound, liver) of mitoxantrone, progesterone, and verapamil 0.28, 1.86, and 25.8 µM, respectively (Table 2).

## 4. Discussion

In vitro stimulation of drug metabolizing enzymes and transporters has long been recognized. However, in vivo or clinical impact of stimulation of drug metabolizing enzymes or transporters are scarce [20,21]. Therefore, the translation of in vitro stimulation to in vivo has not been fully understood. For example, a two-fold stimulation of flurbiprofen metabolism in liver microsome in presence of dapsone yields only 10% increase in flubiprofen clearance in humans, which appears not to be clinically relevant [22,23]. In vitro stimulation of MRP2-mediated transport of different probe substrates, in the presence of a chemically diverse set of compounds, have been widely reported [10,13,14,24]. Previously, we reported that 53% compound of a list of 97 compounds stimulated MRP2-mediated E17βG transport [10]. We found that the stimulation is substrate dependent, as lesser percentage (17%) of compounds stimulated CP-I transport [10]. So far, two previous reports investigated in vivo stimulation of Mrp2- mediated probe substrate transport by measuring the probe compounds, E17βG and GSH, respectively, in rat bile, following treatment with stimulators [16,17]. However, as previously explained, MRP2/Mrp2-mediated E17βG transport is known to be stimulated by many compounds with diverse chemical motifs. We have previously shown that the stimulators used in the study of Heredi-Szabo et al., benzbromarone and indomethacin, are actually inhibitors of MRP2, when CP-I is used as a probe substrate [10]. Therefore, the stimulation effect was re-investigated with CP-I as endogenous biomarker. CP-I, being endogenous, provided us with the advantage that external administration of a radioactive/labelled probe compound could be avoided.

In agreement with the results found in human MRP2 vesicles, rat Mrp2 mediated CP-I transport also displayed conventional Michaelis-Menten kinetics, with Km of 15 µM. The Km values of CP-I in rat Mrp2 vesicles were comparable to that in human (15 vs. 7.7 µM for rat and human MRP2, respectively), suggesting the interspecies differences in affinity of CP-I towards MRP2/Mrp2-meditated biliary secretion is minimum. This is in contrast to E17βG, as significant differences in kinetics of E17βG are reported between different species, such as Michaelis-Menten reported for rat, while sigmoidal kinetics reported for human [14,16]. Four stimulators, mitoxantrone, verapamil, testosterone, and progesterone were selected as tool compounds for the rat in vitro and in vivo investigations, based on their significant stimulation of CP-I transport observed in human MRP2 vesicles and the structural features [10]. All four compounds (structures in supplementary Figure S2) stimulated CP-I transport in membrane vesicles overexpressing rat Mrp2, with mitoxantrone being the strongest stimulator. Mitoxantrone showed a bell-shaped curve: stimulation of Mrp2 mediated CP-I transport at lower concentrations (up to 250 µM) followed by inhibition at higher (>250 µM) concentrations of mitoxantrone (Figure 2). Therefore, two doses of mitoxantrone (6 mg/kg and 15 mg/kg) were administered in bile duct cannulated rats, to assess the impact on CP-I biliary clearance. Mitoxantrone, verapamil (6 mg/kg), and progesterone (1 mg/kg) were investigated by a cross-over study design, so that the drug-pretreatment CP-I and CP-III biliary clearance values can be used as controls to compare with post drug-treatment biliary clearances. Collection of bile was initiated at early time points, as previous reports suggest that the stimulation of Mrp2 mediated transported appeared to occur within the first hour of dosing of indomethacin or benzylpenicillin [16,17].

Contrary to in vitro findings, no significant difference in either biliary clearances or cumulative amount of CP-I or CP-III eliminated in bile were detected before and after treatment of mitoxantrone (Table 1 and Figure 4, Supplementary Table S1). Similarly, neither biliary clearance nor cumulative amount of CP-I excreted of in bile were increased, when dosed with progesterone or verapamil. Although there was a significant increase in CP-I and CP-III levels in bile between 15–30 min, following the 6 mg/kg mitoxantrone dose (Figure 3), the same effect was not observed at the higher dose, nor at following or earlier time intervals in the same dose, suggesting that could be an experimental error. To know if the lack of in vivo translatability of in vitro stimulation is because the compounds are not attaining sufficient concentrations in the liver, free liver concentrations of mitoxantrone, progesterone, and verapamil were determined. In vitro, 1 µM progesterone and 25 µM verapamil stimulated the Mrp2 mediated transport of CP-I to >1.5-fold and >1.3-fold, respectively (Figure 2). The free liver exposures obtained following dosing progesterone and verapamil are 1.86 and 25.8 µM respectively (Table 2). Hence, we can confidently conclude that even after reaching relevant concentrations in liver, progesterone and verapamil did not stimulate Mrp2-mediated CP-I transport in rats. Mitoxantrone dosing was limited by its observed toxicity in rats. Therefore, we could not reach free liver concentration beyond 0.28 µM, while in vitro stimulations started from 1 µM onwards. In vitro, in presence of vesicles, the “free” concentration of the compound is expected to be lower than 1 µM, because of compounds binding to vesicles. Thus, ‘vesicle binding’ will further lower in vitro concentration and we can comfortably assume that “free in vitro concentration” will be in similar range as of “free in vivo concentration”.

Furthermore, apart from direct measurement of CP-I in bile, there are two indirect evidences of not observing any in vivo stimulation of Mrp2 function by mitoxantrone, progesterone, and verapamil. First, the bile flow is not enhanced following compound treatment. Glutathione excretion in bile via Mrp2 mediates bile salt-independent bile flow [25,26]. We assessed the bile flow before and after treatment with respective stimulators and did not observe any increase in bile flow following treatment with the stimulators (Supplementary Table S3). The second indirect proof comes from urinary coproporphyrin ratio (UCP-I/UCP-(I + III)), which is often used as a surrogate marker of MRP2 function and has been used to assess methotrexate PK variability [27]. The urinary coproporphyrin ratio did not decrease following dosing the stimulators (Supplementary Table S4. Collectively, we concluded that the in vitro stimulation of MRP2/Mrp2-mediated CP-I transport is not translatable in vivo.

Plasma and urinary coproporphyrins (CP-I and III) have been suggested as in vivo and clinical biomarkers for organic anion transporting polypeptide (OATP)-mediated drug interactions [28,29,30]. The plasma and urinary levels of CPs are shown to be sensitive markers of Oatp-mediated DDI in cynomolgus monkeys and mice [30]. CP-I uptake in isolated rat hepatocytes was shown to be rifamycin sensitive (Supplementary Figure S1), suggesting a similar role of Oatp transporters in CP dispositions among mice, rats, monkeys, and humans [30]. This opens the possibility of using rats as an investigational model to understand CP-I as an in vivo marker for Oatp inhibition.

In addition to hepatocyte canalicular membranes, Mrp2 is also expressed on the apical side of renal proximal tubules. Thus, one can argue that functional activity of Mrp2 can be monitored via urinary levels of CPs. However, other MRP/Mrp isoforms on the apical side of proximal tubule cells (such as Mrp4), with overlapping substrate specificity, can interfere with Mrp2 function estimation via renal secretion of CPs. Therefore, urinary elimination of CPs is not assessed to understand the in vivo impact of stimulation. In the present investigation, we found the basal plasma levels of CP-III are three-four times higher than CP-I levels, which is similar to what is reported in mice, and is in contrast to monkeys and humans, where CP-I levels are higher in plasma ([30] and Supplementary Table S1). In addition, the biliary clearance of CP-I is about four-fold higher than CP-III, while the urinary clearance of CP-III is three-fold higher than CP-I. This suggests there can be preferential affinity of CP-I towards biliary transporters while CP-III towards urinary transporters. Higher renal clearance of CP-III, compared to CP-I, is observed for monkeys as well [30]. 

To further investigate the possible mechanism for the in vitro-in vivo disconnect of stimulation of CP-I transport via Mrp2, the effect of endogenous compounds such as bile acids and bilirubin on CP-I transport were evaluated. Bile acids did not decrease stimulation by the stimulators (data not shown), while 10 µM bilirubin suppressed the stimulation of Mrp2-mediated CP-I transport to about 50% and 30% of what is observed with 100 and 200 µM mitoxantrone, respectively. To a lesser extent, bilirubin also decreased the stimulation of CP-I transport by 100 µM progesterone and verapamil. Due to the unavailability of bilirubin glucuronides, we were not be able to further assess its effect on the stimulation of Mrp2-mediated CP-I transport by these stimulators. This is the first time that the effect of bilirubin on the in vitro stimulation of Mrp2-mediated transport is reported, to the best of our knowledge. Thus, we hypothesize that endogenous compounds may act on the postulated modulator or transport site of MRP2/Mrp2 and contribute to the in vitro-in vivo discrepancy. This can add to the previous assumptions that the differences in membrane composition between the in vitro and in vivo systems [31] may lead to perceived in vitro to in vivo disconnect in transporter function. The possibility to add bilirubin in vitro to minimize the stimulation effects on MRP2-mediated transport could be further explored to improve the in vitro tools in assessing MRP2 inhibition. Further investigation with altering the in vitro assay can provide confirmatory data in this regard.

## 5. Conclusions

In this work, we first showed that CP-I follows a Michaelis-menten kinetics in rat Mrp2 vesicles. Few compounds (verapamil, mitoxantrone, progesterone, and testosterone) which stimulated MRP2-mediated CP-I transport in human MRP2 vesicles also stimulated rat Mrp2-mediated CP-I transport in vitro. In order to understand the in vivo significance of the ‘stimulation’, bile-duct cannulated rats were dosed with mitoxantrone, verapamil, and progesterone, and CP-I and CP-III levels were measured in bile and urine. Different doses of mitoxantrone, verapamil, or progesterone do not impact Mrp2-mediated CP-I and CP-III excretion in rat bile. This was contradictory to the in vitro data, where mitoxantrone, verapamil, and progesterone stimulated CP-I transport mediated by Mrp2. Our findings support non-existence of any clinical event that can be attributed to stimulation of MRP2-mediated transport. In addition, we also provide evidence in favor of the hypotheses that the presence of an endogenous compound such as bilirubin may lead to the in vitro and in vivo discrepancy.

## Figures and Tables

**Table 1:** Biliary clearances coproporphyrin-I (CP-I) and CP-III in male Sprague Dawley (SD) rats treated with vehicle or modulator.

Dose Group	Modulator Dose (mg/kg, i.v)	Biliary Clearance ^#^ (mL/min)	
		CP-I	CP-III
Vehicle	-	7.6 ± 1.5	2.3 ± 1.3
Mitoxantrone	6	5.9 ± 0.3	1.9 ± 1.7
Vehicle	-	11 ± 0.1	3.3 ± 0.03
Mitoxantrone	15	16 ± 9.5	3.4 ± 2.1
Vehicle	-	14 ± 6.0	2.3 ± 0.2
Progesterone	1	11 ± 4.6	2.3 ± 0.3
Vehicle	-	5.8 ± 2.1	1.6 ± 0.5
Verapamil	10	3.8 ± 0.8	1.2 ± 0.6

^#^: All data values are presented in Mean ± SD, collected from three animal. For experimental design refer to Section 2.4. Saline (0.9% NaCl) was used as vehicle.

**Table 2:** Liver unbound concentrations of modulators.

Dose Group	Modulator Dose (mg/kg, i.v)	In Vivo Parameters ^#^			In Vitro Parameter	Liver Unbound Concentrations ^$^ (µM)
		Liver to Plasma Ratio	C_max _(µM)	Liver Total Concentrations (µM)	F_u,liver_	
Mitoxantrone	6	199	4.3	854	0.0003	0.26
Mitoxantrone	15	202	4.7	950		0.28
Progesterone	1	1.2	165	197	0.0094	1.86
Verapamil	10	63	16.6	1044	0.0247	25.8

**^#^**: Livers were collected at the terminal time point (2 or 3 h) of the respective study, and modulator concentrations were determined in liver homogenate by LC-MS/MS method, refer to Section 2.5 for more details. F_u,liver_: modulator free fraction in liver was determined using rapid equilibrium dialysis method, using blank liver homogenate as matrices. ^$^: Liver unbound concentrations were determined by multiplying F_u,liver _with total liver concentrations.

## Supplementary Materials

The following are available online at http://www.mdpi.com/1999-4923/10/3/125/s1, Figure S1: CP-I uptake in presence and absence of 1 mM rifamycin SV in suspended rat hepatocytes; Figure S2: Structures of the modulators (mitoxantrone, progesterone, testosterone and verapamil) used in this study, along with conventional probe substrate of MRP/Mrp2, estradiol b-glucuronide.; Table S1: Cumulative amount of excretion and area under plasma concentration-time (AUC) profile of CP-I and CP-III following i.v injection of vehicle or modulator.; Table S2: Renal clearance of CP-I and CP-III following i.v injection of vehicle or modulator.; Table S3: Bile flow rate (ml/h) before and after treatment with modulators. The cumulative volume is divided by the total time span of bile collection, to obtain bile flow rate; Table S4: Urinary coproporphyrin (UCP-I/(UCP-I+UCP-III)) ratios with vehicle and compound dosing.

## Abbreviations

E17βG	estradiol-17β-glucuronide
MRP2	multidrug-resistance associated protein 2
OATP/Oatp	organic anion transporting polypeptide
CP-I	coproporphyrin-I
CP-III	coproporphyrin-III LC-MS/MS, liquid chromatography–tandem mass spectrometry
ATP	adenosine tri-phosphate
AMP	adenosine mono-phosphate
ABC	ATP binding cassette
GSH	glutathione
CA	Cholic acid
GCA	Glycocholic acid
TCA	taurocholic acid
DDI	drug-drug interactions
