# Supplementary material for: In Vitro Stimulation of Multidrug Resistance-Associated Protein 2 Function Is Not Reproduced In Vivo in Rats
Source: Pharmaceutics. 2018 Aug 8;10(3):125. doi: 10.3390/pharmaceutics10030125 (PMC6161027; doi:10.3390/pharmaceutics10030125)
Supplement: Supplementary file 1 [file pharmaceutics-10-00125-s001.pdf]

Supplementary Materials: In Vitro Stimulation of Multidrug Resistance-Associated Protein 2 Function is not Reproduced in Vivo in Rats

Ravindranath Reddy Gilibili, Vishwanath Kurawattimath, Bokka Venkata Murali,

Yurong Lai, T. Thanga Mariappan, Hong Shen and Sagnik Chatterjee

Table S1: Cumulative amount of excretion and area under plasma concentration-time (AUC) profile of CP-I and CP-III following i.v injection of vehicle or modular.

Dose group	Modulator dose (mg/kg, i.v)	Cumulative amount excreted in bile (pmol)		Plasma AUC (nM.h)	
		CP-I	CP-III	CP-I	CP-III
Vehicle	-	556 ± 146	1070 ± 368	1.2 ± 0.3	8.2 ± 1.7
Mitoxantrone	6	509 ± 64	1587 ± 726	5.1 ± 6.5	31 ± 31
Vehicle	-	567 ± 42	1043 ± 100	0.9 ± 0.1	5.2 ± 0.5
Mitoxantrone	15	421 ± 97	985 ± 347	0.6 ± 0.5	6.3 ± 3.6
Vehicle	-	672 ± 91	1462 ± 143	0.9 ± 0.3	11 ± 0.3
Progesterone	1	693 ± 256	540 ± 132*	1.1 ± 0.1	3.8 ± 0.6*
Vehicle	-	610 ± 107	1299 ± 332	1.8 ± 0.5	14 ± 2.9
Verapamil	10	485 ± 24	1172 ± 393	2.2 ± 0.4	19 ± 8.2

Data values are presented in mean ± SD collected from three animals. For experimental design refer to Section 2.4. Saline (0.9% NaCl) was used as vehicle. *: Significantly decreased compared to vehicle group ($p < 0.05$).

Table S2: Renal clearance of CP-I and CP-III following i.v injection of vehicle or modulator.

Dose group	Modulator dose (mg/kg, i.v)	Renal clearance # (ml/min)	
		CP-I	CP-III
Vehicle	-	0.8 ± 0.2	2.3 ± 2.3
Mitoxantrone	6	0.9 ± 0.6	3.9 ± 3.9
Vehicle	-	1.5 ± 0.4	6.3 ± 7.1
Mitoxantrone	15	5.6 ± 4.1	27 ± 23
Vehicle	-	1.3 ± 0.6	3.4 ± 0.3
Progesterone	1	2.4 ± 1.4	6.8 ± 3.7
Vehicle	-	1.5 ± 1.5	2.9 ± 2.2
Verapamil	10	0.3 ± 0.04	1.2 ± 0.9

#: All data values are presented in Mean ± SD, collected from three animal. For experimental design refer to Section 2.4. Saline (0.9% NaCl) was used as vehicle.

Table S3: Bile flow rate (ml/h) before and after treatment with modulators. The cumulative volume is divided by the total time span of bile collection, to obtain bile flow rate.

Dose group	Modulator dose (mg/kg, i.v)	Bile Flow # (ml/h)
Vehicle	-	1.32 ± 0.25
Mitoxantrone	6	1.05 ± 0.11
Vehicle	-	1.02 ± 0.17
Mitoxantrone	15	1.38 ± 0.56
Vehicle	-	1.86 ± 0.20
Progesterone	1	1.62 ± 0.39
Vehicle	-	1.41 ± 0.36
Verapamil	10	0.99 ± 0.10

#: All data values are presented in Mean ± SD, collected from three animal. For experimental design refer to Section 2.4. Saline (0.9% NaCl) was used as vehicle.

Table S4: Urinary coproporphyrin (UCP-I/(UCP-I+UCP-III)) ratios with vehicle and compound dosing.

Dose group	Modulator dose (mg/kg, i.v)	Urinary coproporphyrin ratio # [CP-I/(CP-I + CP-II)]
Vehicle	-	0.10 ± 0.11
Mitoxantrone	6	0.08 ± 0.11
Vehicle	-	0.10 ± 0.11
Mitoxantrone	15	0.08 ± 0.11
Vehicle	-	0.02 ± 0.01
Progesterone	1	0.09 ± 0.01
Vehicle	-	0.09 ± 0.11
Verapamil	10	0.08 ± 0.10

#: All data values are presented in Mean ± SD, collected from three animal. For experimental design refer to Section 2.4. Saline (0.9% NaCl) was used as vehicle.

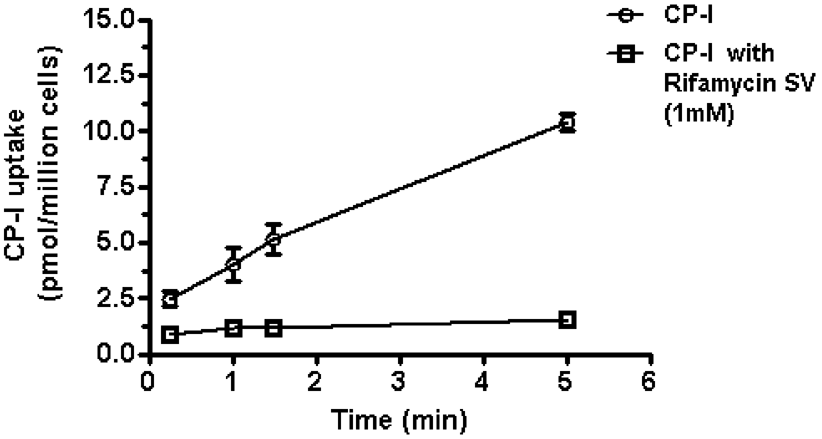

Figure S1: CP-I uptake was performed using silicon oil method. CP-I was tested at 0.5 μM at 37 °C with and without rifamycin SV at 1mM. Samples were collected at 0.25, 1, 1.5 and 5 min. Hepatocyte accumulation of CP-I was analyzed by LC-MS/MS analysis.

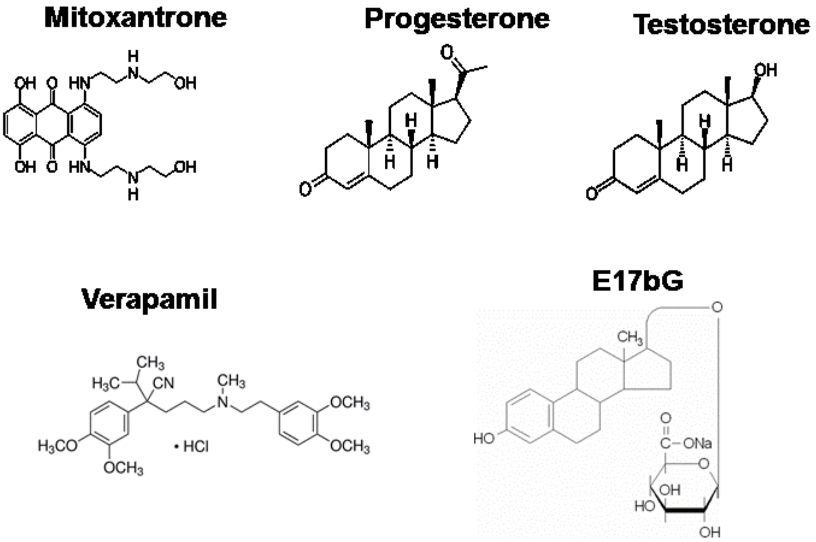

Figure S2: Structures of the modulators (mitoxantrone, progesterone, testosterone and verapamil) used in this study, along with conventional probe substrate of MRP/Mrp2, estradiol β-glucuronide.

Hepatocyte Uptake Experiments

Rat hepatocytes were freshly isolated as per established procedures [30]. For assay purpose, hepatocytes (minimum viability 85%) were diluted with Krebs-Henseleit buffer to obtain 2-million cells/ml. The final assay cell density in hepatocyte suspension was 1-million cells/ml. The uptake assay in hepatocytes was performed as previously described [30]. In brief, hepatocytes were pre-incubated for 3 min at 37.5 °C in a water bath. The assay was started by adding an equal volume of Krebs-Henseleit buffer containing CP-I (final concentration of 1 μM) to the hepatocyte suspension. After stipulated time points, 100 μl of cell mixture was spun down through a silicone and mineral oil layer for 7 sec at 14000 rpm to get the cell pellet at the bottom of the microcentrifuge tube (containing 2 M ammonium acetate in the bottom, on top of it 100 μl of silicone and mineral oil mix with a final density of 1.015). The tubes were immediately transferred onto dry ice to freeze the cell pellet. Following overnight storage of tubes at −80 °C, the cell pellet was collected into 96-well plates by cutting the bottom portion of the tube containing the cell pellet. CP-I was extracted from the cell pellet by a two-step procedure, initially 100 μl of water was added and vortexed at 900 rpm for 1 h and then acetonitrile was added and again vortexed for 1 h. Finally, the supernatant was transferred onto the filter plate attached with a 96-well plate. The filtrate was collected by centrifuging the plate at 4000 rpm for 5 min, and was submitted for LC-MS/MS quantification of CP-I.
